# Caesalpinbondin A, a Novel Diterpenoid Lactone With an Unprecedented Carbon Skeleton from the Seeds of *Caesalpinia bonduc*

**DOI:** 10.3389/fchem.2022.911543

**Published:** 2022-06-24

**Authors:** Dong-Qing Fei, Hui-Hong Li, Xiao-Han Chen, Wen-Bo Cui, Zong-Ping Zhang, Xiao-Qing Zhan, Mei-Jie Wang, Feng-Ming Qi, Zhan-Xin Zhang, Er-Wei Li

**Affiliations:** ^1^ School of Pharmacy, State Key Laboratory of Applied Organic Chemistry, Lanzhou University, Lanzhou, China; ^2^ Translational Medicine Center, Zhengzhou Central Hospital Affiliated to Zhengzhou University, Zhengzhou, China; ^3^ Institutional Center for Shared Technologies and Facilities, Institute of Microbiology, Chinese Academy of Sciences, Beijing, China; ^4^ State Key Laboratory of Mycology, Institute of Microbiology, Chinese Academy of Sciences, Beijing, China

**Keywords:** Fabaceae, *Caesalpinia bonduc*, diterpenoid, caesalpinbondin A, *Caenorhabditis elegans*, anti-Alzheimer’s disease bioactivity

## Abstract

One novel diterpenoid lactone named caesalpinbondin A (**1**) that possesses an unprecedented tetracyclic ring system in which a 6/6/5-fused tricyclic ring and a 4,5-dimethyldihydrofuran-2(3H)-one were connected by a C-C single bond comprising a 5-(naphtho [2,3-b]furan-7-yl)dihydrofuran-2(3H)-one moiety was isolated from the seeds of *Caesalpinia bonduc*. Its chemical structure was established by extensive spectroscopic methods, and its absolute configuration was further determined by single-crystal X-ray diffraction analysis and electronic circular dichroism calculation. The biological evaluation suggested that compound **1** demonstrated potent anti-Alzheimer’s disease (AD) bioactivity, which could delay paralysis of transgenic AD *Caenorhabditis elegans*. A possible biogenetic pathway of **1** was also proposed.

## Graphical Abstract

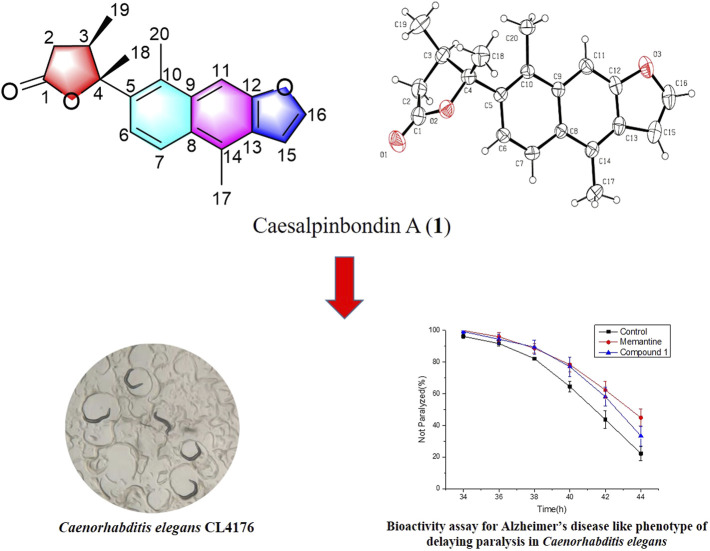

## Introduction

Between 1981 and 2019, approximately 50% of 1,881 new drugs approved worldwide were produced using or inspired by natural products or derivatives (Newman and Cragg, 2020). In particular, natural products, which are derived from medicinal plants with new types of architectures and amazing bioactivities, have been a rich source of drug leads and candidates.

The genus *Caesalpinia*, belonging to the Fabaceae family, comprises approximately 100 different kinds of plants that are mainly distributed in tropical and subtropical regions including China, Thailand, Vietnam, and other countries (Qiao et al., 2016). There are 17 *Caesalpinia* species in China, most of which are found in the southern regions (Wei et al., 2016). Many plants of the *Caesalpinia* genus have been used as a traditional medicine to treat multiple diseases in many countries all over the world (Wu et al., 2011). The chemical constituents from this genus have been reported to be terpenoids, flavonoids, steroids, lignans, phenylpropanoids, and alkaloids (Wu et al., 2011; Zanin et al., 2012). Among these constituents, the cassane-type diterpenoids, the main and characteristic chemical components of *Caesalpinia*, have been studied widely due to their bioactivities, such as anti-inflammatory, antitumor, antimalarial, antiviral, antioxidant, and antimicrobial properties (Jing et al., 2019). Furthermore, some diterpenoids with novel skeletons have been found in plants of the *Caesalpina* genus, such as caesalpinimin A (Zhang et al., 2014), spirocaesalmin (Jiang et al., 2001), tomocinol C, and spirocaesalmin C (Xiao et al., 2016).

*Caesalpinia bonduc* (Linn.) Roxb. is a famous medicinal plant that grows mainly in tropical and subtropical regions of Southeast Asia (Pudhom et al., 2007). In China, it is mainly distributed in the southern areas, such as Guangxi, Hainan, and Taiwan (Editorial Committee of Flora of China, 1998). The seeds of *C. bonduc* have been used as a traditional Chinese medicine (TCM) for the treatment of a variety of diseases, including common cold, fever, dysentery, epigastric pain, abdominal pain, eye swelling and pain, and sores (Jiangsu New Medical College, 1977; Editorial Board of Chinese Materia Medica, 1999). Extracts or constituents of *C. bonduc* have been reported to possess cytotoxicity (Wu et al., 2014), antiproliferative (Yadav et al., 2009), anti-inflammatory (Liu et al., 2020), antistress (Kannur et al., 2006), antimicrobial (Simin et al*.*, 2001; Arif et al., 2009), and anti-insecticidal effects (Essoung et al., 2020). Previous phytochemical investigations of *C. bonduc* have demonstrated the presence of cassane-type diterpenoids (Wu et al., 2014; Zhang et al., 2016; Liu et al., 2020; Cao et al., 2021), sterols (Udenigwe et al., 2007), and flavonoids (Ata et al., 2009). Among these chemical constituents, cassane-type diterpenoids were found to be the most important components and were considered to be active constituents of *C. bonduc*.

**FIGURE 1:**
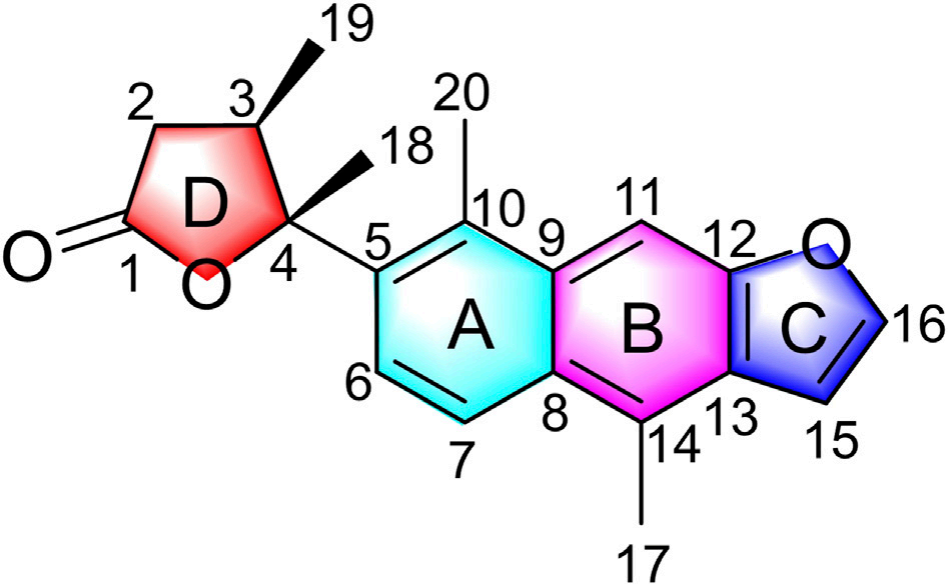
Structure of caesalpinbondin A (**1**).

In our continuing search for structurally novel and biologically interesting natural products of Chinese medicinal plants (Fei et al., 2016; Li et al., 2017; Wu et al., 2018; Zhang et al., 2018; Yu et al., 2020; Li et al., 2022), a novel diterpenoid, caesalpinbondin A (**1**) (Figure 1), was isolated from the 95% ethanol extract of the seeds of *C. bonduc*. Compound 1 represented a novel tetracyclic ring system in which a 6/6/5-fused tricyclic ring and a 4,5-dimethyldihydrofuran-2(3H)-one moiety were connected by a C-C single bond comprising an unprecedented 5-(naphtho [2,3-b]furan-7-yl)dihydrofuran-2(3H)-one scaffold. We present the isolation, structure and stereochemistry elucidation, plausible biosynthetic pathway, and anti-AD activity of **1**.

## Results and Discussion

The 95% EtOH extract of the seeds of *C. bonduc* was partitioned by successive extraction with EtOAc and *n*-BuOH. The EtOAc-soluble fraction was separated by repeated column chromatography on silica gel and Sephadex LH-20 to afford compound **1**.

Compound **1** was obtained as colorless needle-like crystals with [α]^20^_D_ +14.0 (*c* 0.10, CHCl_3_). Its IR spectrum showed absorption bands assignable to carbonyl (1773 cm^−1^) and aromatic ring (1,607 and 1,500 cm^−1^) functionalities. The molecular formula of C_20_H_20_O_3_, with 11 degrees of unsaturation, was deduced from HRESIMS at *m/z* 331.1310 [M + Na]^+^ (calcd for C_20_H_20_O_3_Na, 331.1305), which was supported by the NMR data (Table 1). The ^1^H NMR spectrum displayed signals of five aromatic protons at *δ*_H_ 7.60 (1H, d, *J* = 9.6 Hz, H-6), 8.00 (1H, d, *J* = 9.6 Hz, H-7), 8.05 (1H, s, H-11), 6.94 (1H, d, *J* = 2.4 Hz, H-15), and 7.72 (1H, d, *J* = 2.4 Hz, H-16); two benzylic angular methyls at *δ*_H_ 2.87 (3H, s, H-17) and 2.79 (3H, s, H-20); one secondary methyl at *δ*_H_ 1.32 (3H, d, *J* = 7.2 Hz, H-19); and one tertiary methyl at *δ*_H_ 1.82 (3H, s, H-18). In addition, the ^1^H NMR spectrum showed resonances due to one aliphatic methine at *δ*_H_ 3.08 (1H, m, H-3) and one aliphatic methylene at *δ*_H_ 2.18 (1H, dd, *J* = 18.0, 3.0 Hz, H-2a) and 2.59 (1H, dd, *J* = 18.0, 3.0 Hz, H-2b). The ^13^C and DEPT NMR spectra revealed 20 resonances ascribed to four methyls, one methylene, six methines (five olefinic and one aliphatic), and nine quaternary carbons (one carbonyl, seven olefinic, and one oxygenated aliphatic). Among them, one carbonyl and twelve sp^2^-hybridized carbons indicated seven degrees of unsaturation. These data suggested that compound **1** is a diterpenoid possessing a tetracyclic ring system in the scaffold.

**TABLE 1:** ^1^H (600 MHz) and^13^C NMR (150 MHz) spectral data of compound **1** in CDCl_3_ (*δ* in ppm, *J* in Hz).

No.	*δ*_C_	*δ*_H_ Mult (*J*, hz)	HMBC
1	176.4, qC	—	—
2	37.2, CH_2_	2.59, dd (18.0, 3.0)	1, 3, 4, and 19
	—	2.18, dd (18.0, 3.0)	—
3	39.4, CH	3.08, m	1, 2, 4, 5, 18, and 19
4	90.4, qC	—	—
5	138.6, qC	—	—
6	120.2, CH	7.60, d (9.6)	4, 5, 7, 8, and 10
7	122.7, CH	8.00, d (9.6)	5, 6, 8, 9, and 14
8	128.2, qC	—	—
9	132.3, qC	—	—
10	129.4, qC	—	—
11	101.6, CH	8.05, s	8, 9, 10, 12, and 13
12	153.9, qC	—	—
13	127.8, qC	—	—
14	126.4, qC	—	—
15	105.2, CH	6.94, d (2.4)	12, 13, 14, and 16
16	146.9, CH	7.72, d (2.4)	12, 13, and 15
17	15.6, CH_3_	2.87, s	8, 13, and 14
18	23.5, CH_3_	1.82, s	3, 4, and 5
19	16.4, CH_3_	1.32, d (7.2)	2, 3, and 4
20	17.9, CH_3_	2.79, s	5, 9, and 10

**FIGURE 2:**
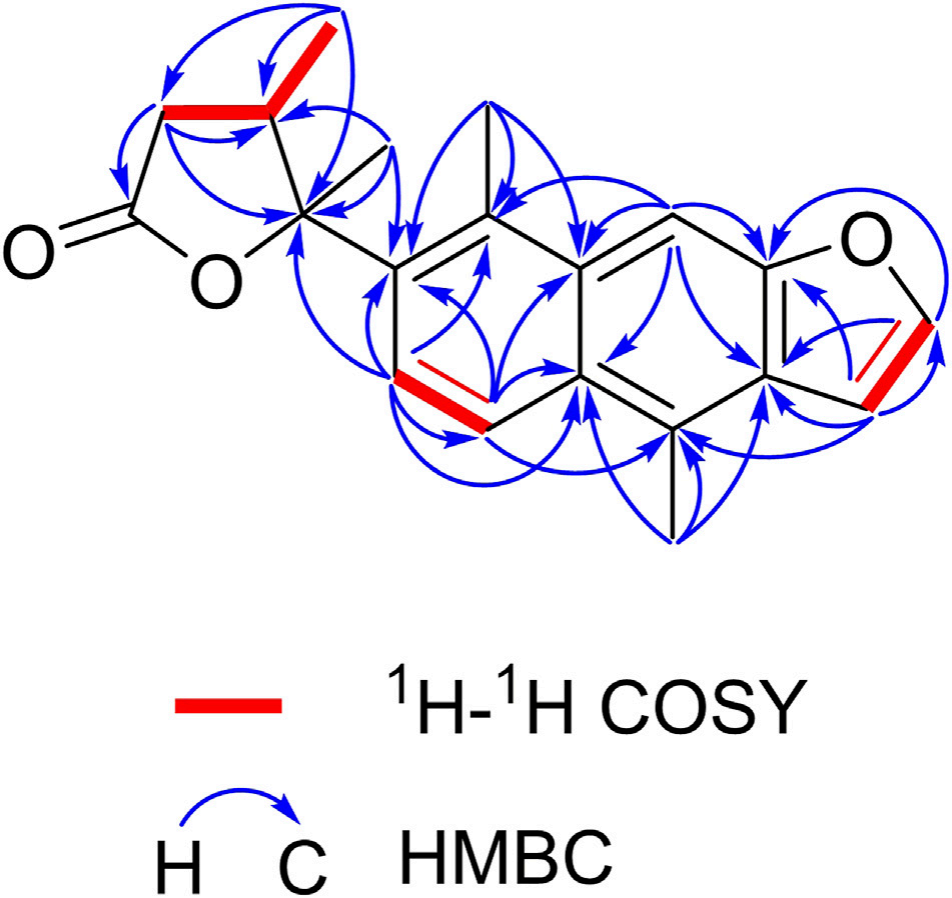
Key ^1^H–^1^H COSY and HMBC correlations of caesalpinbondin A (**1**).

The planar structure of **1** was elucidated by extensive analysis of the two-dimensional (2D) NMR experiments. The proton and proton-bearing carbon resonances in the NMR spectra of **1** were assigned unambiguously by comprehensive interpretation of the ^1^H–^1^H COSY (Figure 2) and HSQC spectroscopic data. The proton spin–spin coupling system from H-6 to H-7 in the ^1^H–^1^H COSY spectrum, in combination with the HMBC correlations (Figure 2) from H_3_-20 to C-5, C-9, and C-10, from H-6 to C-5, C-7, C-8, and C-10, from H-7 to C-5, C-6, C-8, and C-9, demonstrated the presence of a benzene ring A with a methyl group at C-10. Moreover, the HMBC correlations of H_3_-17 with C-8, C-13, and C-14 and of H-11 with C-8, C-9, C-10, C-12, and C-13 indicated the existence of a benzene ring B fused with ring A through the positions between C-8 and C-9 with a methyl group at C-14. Subsequently, the ^1^H–^1^H COSY correlation of H-15 with H-16, the HMBC correlations of H-15 with C-12, C-13, C-14, and C-16, and the HMBC correlations of H-16 with C-12, C-13, and C-15, in combination with the dramatically downfield chemical shifts of C-12 (*δ*_C_ 153.9) and C-16 (*δ*_C_ 146.9), suggested the existence of a furan ring C fused with ring B through the positions between C-12 and C-13. Additionally, the ^1^H–^1^H COSY correlations of H_2_-2/H-3/H_3_-19, as well as the HMBC cross-peaks of H_2_-2 with C-1, C-3, and C-4, of H-3 with C-1, C-2, and C-4, and of H_3_-19 with C-2, C-3, and C-4, revealed that the carbonyl C-1 of **1** (*δ*_C_ 176.4) formed a γ-butyrolactone (ring D) with the oxygenated C-4 (downfield chemical shift at *δ*_C_ 90.4) to occupy the remaining one degree of unsaturation. The HMBC correlations of H_3_-19 with C-2, C-3, and C-4 and of H_3_-18 with C-3 and C-4 indicated that CH_3_-19 and CH_3_-18 were located at C-3 and C-4, respectively. Based on the HMBC correlations of H_3_-18 with C-5 and of H-6 with C-4, it was further apparent that the ring D and ring A were linked between C-4 and C-5 by a C-C single bond.

**FIGURE 3:**
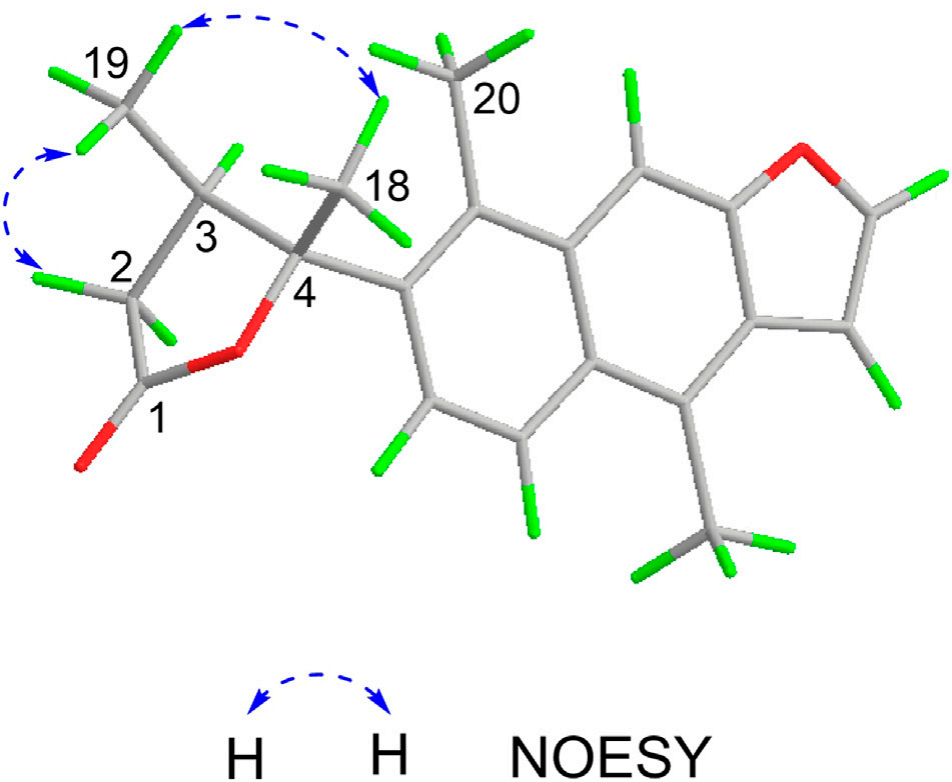
Key NOESY correlations of caesalpinbondin A (**1**).

**FIGURE 4:**
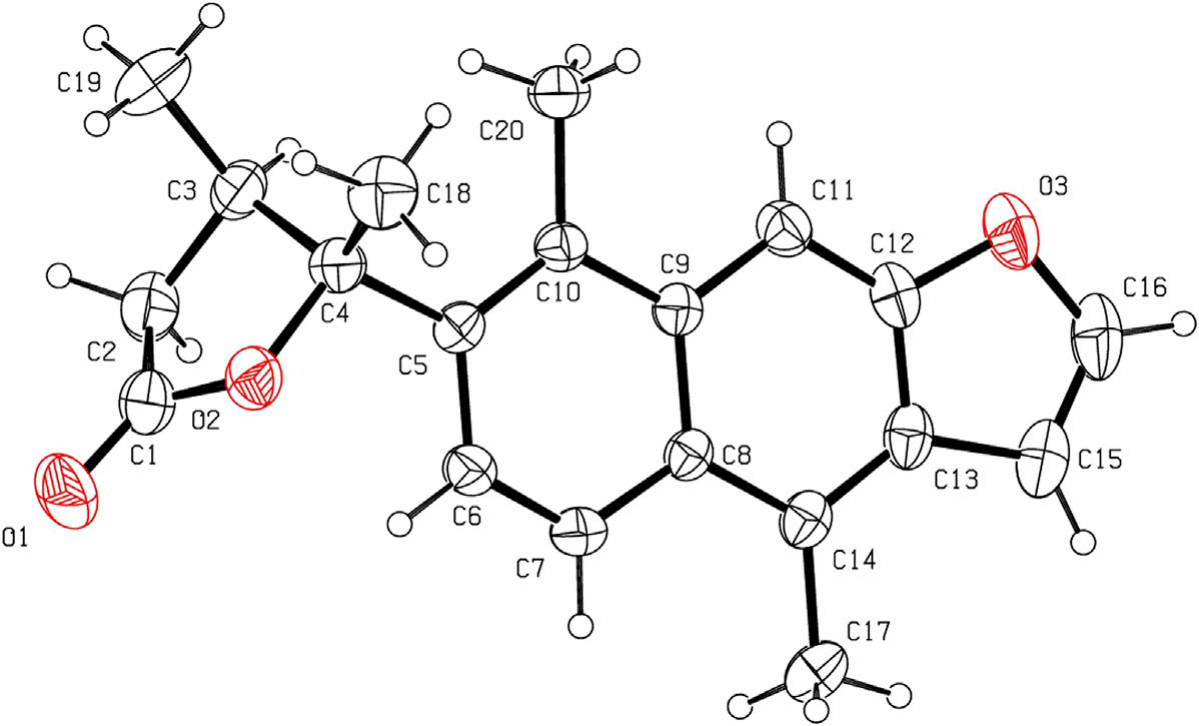
ORTEP plot of the X-ray crystallographic structure for **1** (displacement ellipsoids were drawn at the 30% probability level).

**FIGURE 5:**
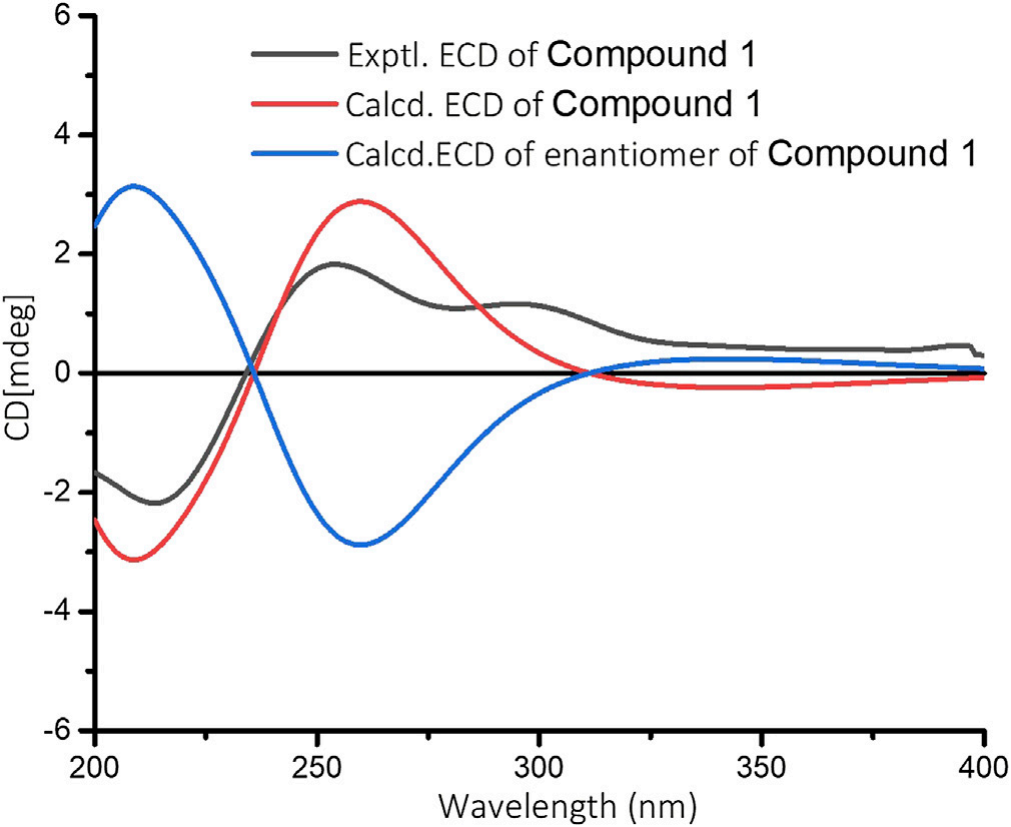
Experimental ECD spectrum of **1** and calculated ECD spectra for (3*R*, 4*R*)-**1** and its enantiomer in MeOH.

The relative configuration of **1** was determined by NOESY analysis. In the NOESY spectrum (Figure 3), the NOE correlation between H_3_-18 and H_3_-19 indicated that H_3_-18 and H_3_-19 were oriented on the same side of the γ-butyrolactone moiety (*β*-configuration). Thus, the planar structure and relative configuration of **1** were determined as shown in Figure 1. After many attempts to crystallize **1** using different solvents, a single crystal of **1** was finally obtained from CHCl_3_-MeOH (2:3). The subsequent X-ray crystallographic analysis (Cu Kα) clarified the planar structure and the relative configuration of **1** (Figure 4). However, the Flack parameter (-0.3 (3)) was somewhat large, precluding assignment of the absolute configuration of **1** by this method. Thus, ECD calculation was utilized to identify the absolute configuration of **1**. Using quantum-mechanical time-dependent density functional theory (TDDFT) calculation, the theoretically calculated ECD spectrum of **1** (Figure 5) was in good agreement with the experimental ECD spectrum, supporting the assignment of the absolute configuration of **1** as 3 and 4*R*. Thus, the structure of **1** was established and named caesalpinbondin A.

Caesalpinbondin A (**1**) possessed an unconventional tetracyclic diterpenoid backbone, in which a 6/6/5-fused tricyclic ring system was connected to a 4,5-dimethyldihydrofuran-2(3H)-one moiety *via* a C-C single bond. The aforementioned analyses proved **1** as a diterpenoid with a new carbon skeleton architecture, and the name “caesalpinane” is suggested for this new skeleton type.

**SCHEME 1:**
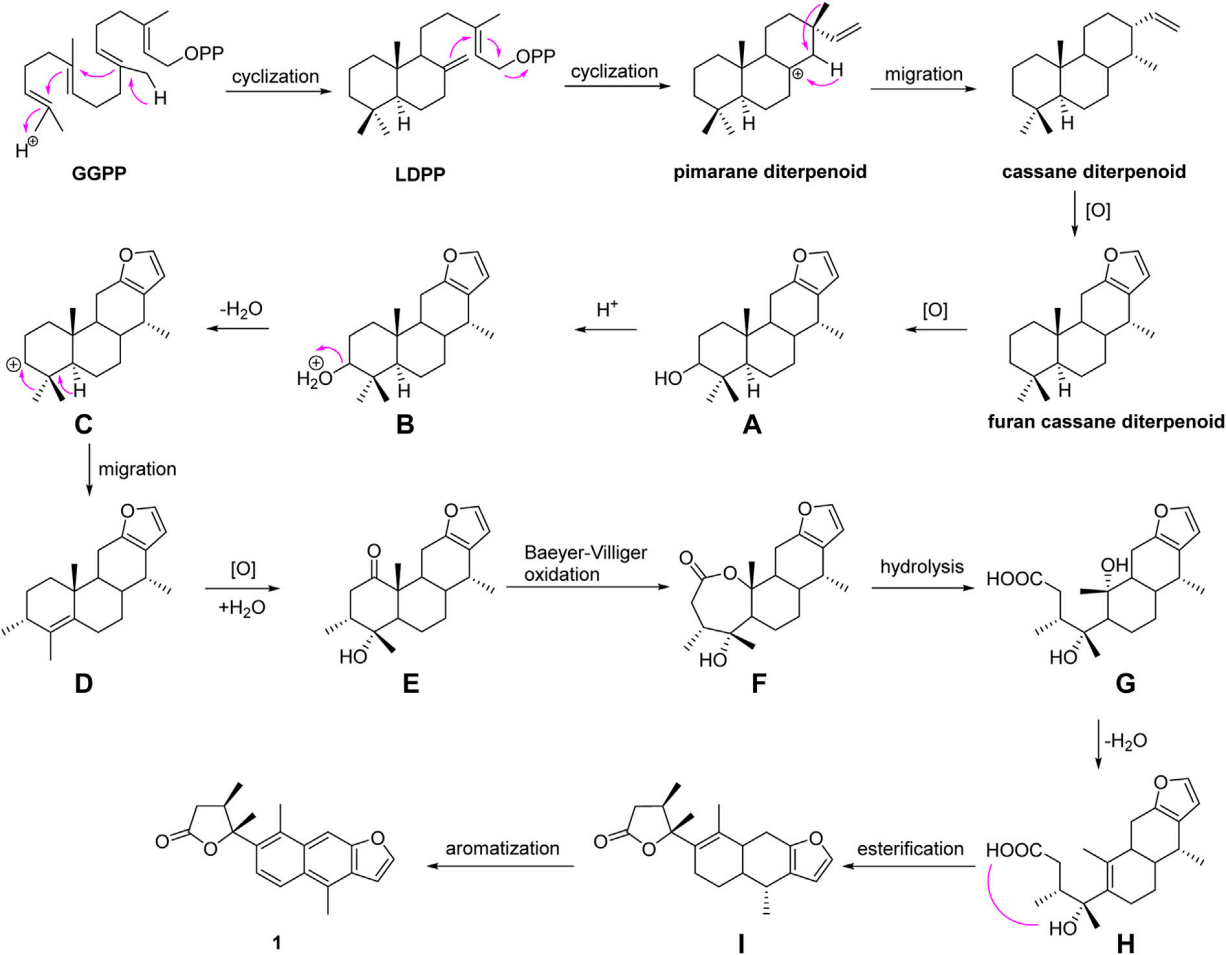
Plausible biosynthetic pathway for **1**.

A hypothetical biosynthetic route was proposed, as shown in Scheme 1. Compound **1** should be originated from geranylgeranyl pyrophosphate (GGPP), which is a typical precursor of diterpenoids. First, labdadienyl pyrophosphate (LDPP) was produced from GGPP by an acid-induced intramolecular cyclization, and then the pimarane-type diterpenoid cation intermediate was generated from LDPP through further intramolecular cyclization and converted to the cassane-type diterpenoid *via* the migration of methyl 17 from C-13 to C-14 (Zentar et al., 2018; Jing et al., 2019). The cassane-type diterpenoid was transformed into furan-cassane diterpenoid through oxidation, nucleophilic ring closure, and dehydration reactions (Zhang et al., 2013). Next, compound **A** could be derived from the furan-cassane diterpenoid *via* oxidation reaction and further hydrogenated and dehydrated to afford a key carbocation intermediate **C**, which could be converted to **D** by the migration of methyl 19 from C-4 to C-3. Subsequent oxidation and addition of compound **D** may lead to the formation of compound **E**, which could go through Baeyer–Villiger oxidation to give compound **F**. Hydrolysis of compound **F** followed by dehydrated and intramolecular esterification reaction could produce compound **I**, which would give compound **1** by an enzyme-mediated aromatization.

**FIGURE 6:**
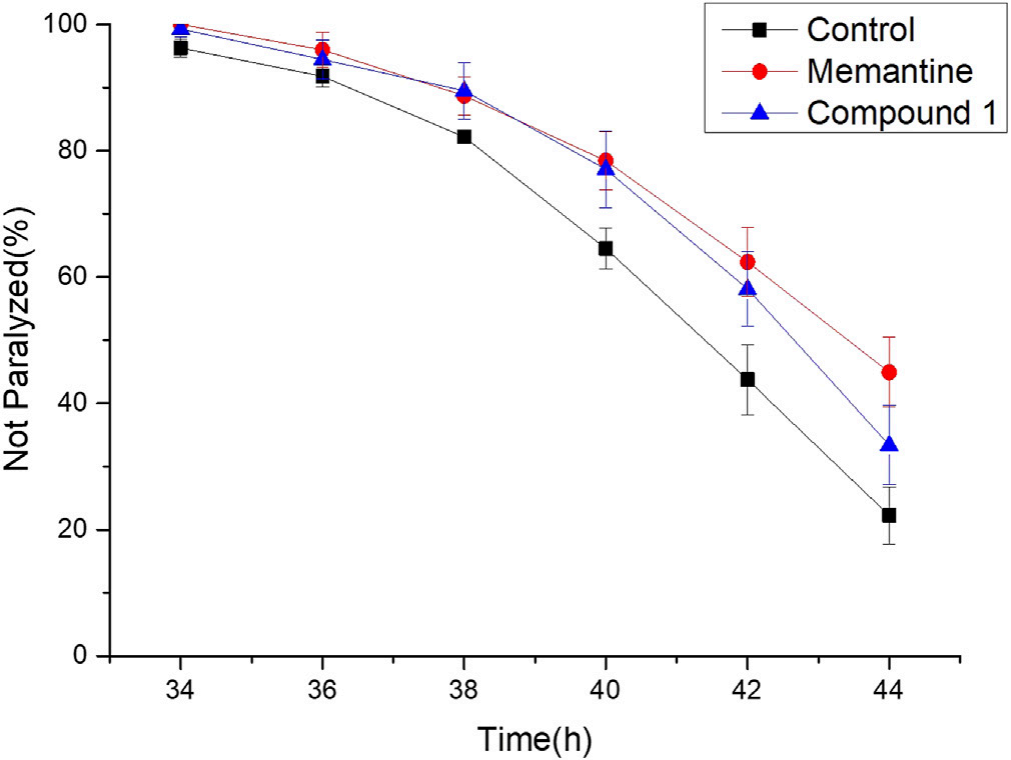
Anti-Alzheimer’s disease (AD) activity of compound **1**, positive control, and control. Worms were treated with 100 μM compound **1**, 100 μM memantine was regarded as the positive control, and 0.1% DMSO was regarded as the control.

Furthermore, the anti-Alzheimer’s disease (AD) bioactivity of compound **1** was evaluated using the transgenic AD *Caenorhabditis elegans* model with memantine as the positive control (Figure 6). The AD model (CL4176) is based on the transfer of the human Aβ_1-42_ gene downstream of the nematode *myo-3* promoter. When incubated at a normal temperature of 15°C, the nematodes did not express Aβ_1-42_, and when the temperature was adjusted upward to 25°C, Aβ_1-42_ began to accumulate in the muscle cells and induced a toxic response that paralyzed the nematodes. If the number of nematode individuals in the paralyzed state was reduced in the administered group compared to the blank group, the compound was shown to have anti-AD activity. For its cheapness, easy and fast-breeding, and AD pathological similarity, the transgenic AD *C. elegans* model has been used as a powerful tool for rapid screening of anti-AD drugs (Xin et al., 2013). Using transgenic AD *C. elegans* paralysis assay, we have successfully discovered several natural products with potential anti-AD effects (Li et al., 2017; Wu et al., 2018; Su et al., 2021). As shown in Figure 6, compound **1** significantly delayed the Aβ-induced paralysis in CL4176 nematodes (*p* < 0.05). At 44th h of upregulated temperature induction, 33.40% of nematodes were still unparalyzed in the 100 μM compound **1**-treated group, while only 22.24% of unparalyzed nematodes remained in the 0.1% DMSO-treated group. The result of the bioactivity assay indeed gave a notion that compound **1** may have the potential to be served as an anti-AD agent candidate.

## Conclusion

In summary, our further exploration of the minor chemical constituents from *C. bonduc* yielded one new carbon skeleton diterpenoid, caesalpinbondin A (**1**). The novel diterpenoid **1** possessed an unprecedented 6/6/5-fused ring system with a 4,5-dimethyldihydrofuran-2(3H)-one moiety scaffold. The discovery of **1** has enriched the diversity and complexity of natural diterpenoids. In addition, compound **1** exhibited good delayed AD-like symptoms of worm paralysis phenotype activity, suggesting that this compound may be useful for the development of anti-AD drugs. These findings will attract much attention from both chemists and pharmacologists, and further research such as total synthesis and in-depth pharmacological tests are warranted.

## Experimental Section

### General Experimental Procedures

Melting points were measured using an X-4 digital display micromelting point apparatus (Beijing Tech Instrument Co., Ltd. Beijing, China) and are uncorrected. Optical rotations were obtained on a PerkinElmer 341 polarimeter (PerkinElmer, Waltham, MA, United States). IR spectra were run on a Nicolet NEXUS 670 FT-IR spectrometer (ThermoFisher Scientific Inc. Waltham, MA, United States). The ECD spectra were measured on an OLIS DSM1000 spectrophotometer (Olis, Inc. Bogart, GA, United States) at room temperature. The 1D and 2D NMR experiments were conducted on a Varian INOVA 600 MHz NMR spectrometer (Varian Inc. Palo Alto, CA, United States) and used TMS as the internal standard. The HRESIMS data were collected on a ThermoFisher LTQ Orbitrap Elite high-resolution mass spectrometer (Thermo Fisher Scientific Inc. Waltham, MA, United States). The X-ray crystallographic analysis was performed on an Agilent SuperNova dual-wavelength diffractometer with a microfocus X-ray source and multilayer optics monochromatized Cu Kα (*λ* = 1.54184 Å) radiation (Agilent Technologies, Santa Clara, CA, United States). Silica gel (200–300 mesh (Qingdao Marine Chemical Factory, Qingdao, China) and Sephadex LH-20 (GE Healthcare Amersham Biosciences, Uppsala, Sweden) for column chromatography were used to separate compounds. Silica gel GF254 (10–40 *μ*m) used for TLC was supplied by Qingdao Marine Chemical Factory, Qingdao, China. Spots were detected on TLC under UV light or by heating after spraying with 5% H_2_SO_4_ in C_2_H_5_OH (v/v).

### Plant Material

The seeds of *C. bonduc* were purchased in April 2014 from the Anguo Traditional Chinese Medicine Market in Hebei Province, China, and identified by Dr. Jian-yin Li of School of Pharmacy, Lanzhou University. A voucher specimen (No. 20140418CB) was deposited at the School of Pharmacy, Lanzhou University, China.

### Extraction and Isolation

The air-dried and powdered seeds of *C. bonduc* (12 kg) were extracted with 95% EtOH (40 L × 4) at room temperature. Evaporation of the organic solvent afforded 920 g of a crude extract, which was suspended in water and successively partitioned into EtOAc and *n*-BuOH extracts. The EtOAc extract (300 g) was separated over a silica gel column with a petroleum ether/acetone gradient (from 40:1 to 0:1, v/v) eluent to provide seven fractions (Fr.1–Fr.7). Fraction 2 (25 g) was chromatographed over silica gel eluted with petroleum ether/EtOAc (from 30:1 to 3:1, v/v) to yield four subfractions (Fr.2A–Fr.2D). Fr.2C (900 mg) was subjected to passage over a silica gel column with petroleum ether/acetone (from 20:1 to 0:1. v/v) and then applied to Sephadex LH-20 column chromatography (CHCl_3_/CH_3_OH, 2:3, v/v) to obtain **1** (5 mg).

### Physical and Chemical Data

Caesalpinbondin A (**1**): colorless needle-like crystals; m. p. 218–220°C [α]^20^_D_ +14.0 (*c* 0.10, CHCl_3_); IR (KBr) ν_max_: 2,974, 2,926, 1773, 1,638, 1,607, 1,543, 1,500, 1,459, 1,385, 1,231, 1,142, 1,045, 934, 756, and 666 cm^−1^; ^1^H (600 MHz) and ^13^C (150 MHz) NMR data, see Table 1; HRESIMS *m/z* 331.1310 [M + Na]^+^ (calcd. for C_20_H_20_O_3_Na, 331.1305).

### Single X-Ray Diffraction Data Analysis

Single crystals of compound **1** was obtained by recrystallization from a mixture of CHCl_3_ and MeOH. Crystal data for **1**: C_20_H_20_O_3_, *M*r = 308.36, orthorhombic, space group *P*2_1_2_1_2_1_, *a* = 6.26793 (18) Å, *b* = 12.7202 (4) Å, *c* = 20.3959 (6) Å, *α* = 90.00°, *β* = 90.00°, *γ* = 90.00°, *V* = 20.3959 (6) Å^3^, *T* = 293 (2) K, *Z* = 4, *d* = 1.260 g/cm^3^, *μ*(Cu Kα) = 0.67 mm^−1^, *F* (000) = 656, and crystal dimensions 0.24 × 0.09 × 0.07 mm were used for measurement on an Agilent Technologies SuperNova, Dual source, EOS CCD with mirror optics, using graphite monochromated Cu K*α* radiation (*λ* = 1.54184 Å). The total of 13,844 reflections measured 3,009 independent reflections (*R*_int_ = 0.0371). The final *R*_1_ value was 0.0428 (*I* > 2σ (*I*)). The final w*R* (*F* ^2^) value was 0.1116 ((*I* > 2σ(*I*)). The final *R*_1_ value was 0.0610 (all data). The final w*R* (*F* ^2^) value was 0.0972 (all data). The goodness of fit on *F* ^2^ was 1.031. The Flack parameter was -0.3 (3). Crystallographic data for the structure of compound **1** have been deposited with the Cambridge Crystallographic Data Centre (deposition no. CCDC 2106390). Copies of these data can be obtained free of charge *via* www.ccdc.cam.ac.uk/conts/retrieving.html (or from the Cambridge Crystallographic Data Centre, 12 Union Road, Cambridge CB21EZ, U.K.; fax (+44) 1223-336-033; or deposit@ccdc.cam.uk).

### ECD Calculations

The conformational search for the enantiomers of all plausible stereoisomers of **1** was performed by the “random search” procedure implemented in the SYBYL-X-2.1.1 program using the Molecular Merck force field (MMFF94s). Gaussian 09 software was applied to screen stable conformers with the energy of the optimized structures at the B3LYP/6-31G(d) level (Frisch et al., 2010). The ECD curves of the conformers were determined by the TDDFT method at the B3LYP/6-31+G(d) level with the CPCM model in a methanol solution. SpecDis 1.7.1 software with UV correction was used to weigh the overall ECD curves by the Boltzmann distribution of each conformer (Bruhn et al., 2017). The calculated ECD curves of **1** were compared with the experimental results for the absolute configuration determination.

### Bioactivity Assay for Alzheimer’s Disease-Like Phenotype of Delaying Paralysis in *Caenorhabditis elegans*

The genetically modified *Caenorhabditis elegans* CL4176 strain with genotype smg-1 [myo-3p:: A-Beta (1–42):: let-851 3′UTR) + rol-6 (su1006)] was purchased from the *Caenorhabditis* Genetics Center (CGC) (University of Minnesota, Minneapolis, MN). Using *Escherichia coli* OP50 as the standard food source, worms were cultured on nematode growth medium (NGM) at 16°C. A total of 60–80 eggs were placed in NGM, and 0.1% DMSO was used as a negative control for the entire biological activity determination experiment. After the larvae developed into L3 larvae, they were transferred to another 25°C incubator and incubated for 26 h. The paralyzed worms were observed and counted under a dissecting microscope every 2 h until they were all paralyzed. The non-paralysis curve was plotted by incubation time at 25°C as *x*-axis and non-paralytic worm percentage as *y*-axis throughout the experiment. The compound delaying the rate of worm paralysis more effectively means that it has higher anti-AD activity than any others. It can be identified that the non-paralysis curve of a compound with a higher anti-AD activity locates on the topside of the other inferior. The anti-AD activity was determined by using a log-rank survival test to compare the significance between treatments (Martorell et al., 2013). The *p-*value at a level of 0.05 or less was considered to be statistically significant.

## Data Availability

The original contributions presented in the study are publicly available. These data can be found at: https://www.ccdc.cam.ac.uk/, 2106390.
