# Supplementary material for: Caesalpinbondin A, a Novel Diterpenoid Lactone With an Unprecedented Carbon Skeleton from the Seeds of Caesalpinia bonduc
Source: Front Chem. 2022 Jun 24;10:911543. doi: 10.3389/fchem.2022.911543 (PMC9263540; doi:10.3389/fchem.2022.911543)
Supplement: Supplementary file 1 [file DataSheet1.docx]

Supplementary Material

**Caesalpinbondin A, a Novel Diterpenoid Lactone With an Unprecedented Carbon Skeleton from the Seeds of** ***Caesalpinia bonduc***

**Dong-Qing Fei^1*^, Hui-Hong Li^1,4^, Xiao-Han Chen^1^, Wen-Bo Cui^1^, Zong-Ping Zhang^1^, Xiao-Qing Zhan, Mei-Jie Wang, Feng-Ming Qi^1^, Zhan-Xin Zhang^1*^ and Er-Wei Li^2,3*^**

^1^ School of Pharmacy, State Key Laboratory of Applied Organic Chemistry, Lanzhou University, Lanzhou, China

^2^ Institutional Center for Shared Technologies and Facilities, Institute of Microbiology, Chinese Academy of Sciences, Beijing, China

^3^ State Key Laboratory of Mycology, Institute of Microbiology, Chinese Academy of Sciences, Beijing, China

^4^ Translational Medicine Center, Zhengzhou Central Hospital Affiliated to Zhengzhou University, Zhengzhou, China

*** Correspondence:**Dong-Qing Fei
[feidq@lzu.edu.cn](mailto:feidq@lzu.edu.cn)

Zhan-Xin Zhang
[zhangzhx@lzu.edu.cn](mailto:zhangzhx@lzu.edu.cn)

Er-Wei Li
[liew@im.ac.cn](mailto:liew@im.ac.cn)

**TABLE OF CONTENTS**

[**Figure S1.** ^1^H NMR spectrum of caesalpinbondin A (**1**) in CDCl_3_ 3](#_Toc101732178)

[**Figure S2.** ^13^C NMR and DEPT spectra of caesalpinbondin A (**1**) in CDCl_3_ 4](#_Toc101732179)

[**Figure S3.** HSQC spectrum of caesalpinbondin A (**1**) in CDCl_3_ 5](#_Toc101732180)

[**Figure S4.** HMBC spectrum of caesalpinbondin A (**1**) in CDCl_3_ 6](#_Toc101732181)

[**Figure S5.** ^1^H-^1^H COSY spectrum of caesalpinbondin A (**1**) in CDCl_3_ 7](#_Toc101732182)

[**Figure S6.** NOESY spectrum of caesalpinbondin A (**1**) in CDCl_3_ 8](#_Toc101732183)

[**Figure S7.** HRESIMS spectrum of caesalpinbondin A 9](#_Toc101732184)

[**Figure S8.** IR spectrum of caesalpinbondin A 10](#_Toc101732185)

[**Figure S9.** UV spectrum of caesalpinbondin A (MeOH) 11](#_Toc101732186)

[**Figure S10.** Experimental ECD spectrum of caesalpinbondin A (MeOH) 12](#_Toc101732187)

[**Figure S11.** Energy lowest conformers and populations of (3*R*,4*R*)-**1** and optimized coordinates (method: B3LYP/6-31G) 13](#_Toc101732188)

**Figure S1.** ^1^H NMR spectrum of caesalpinbondin A (**1**) in CDCl_3_


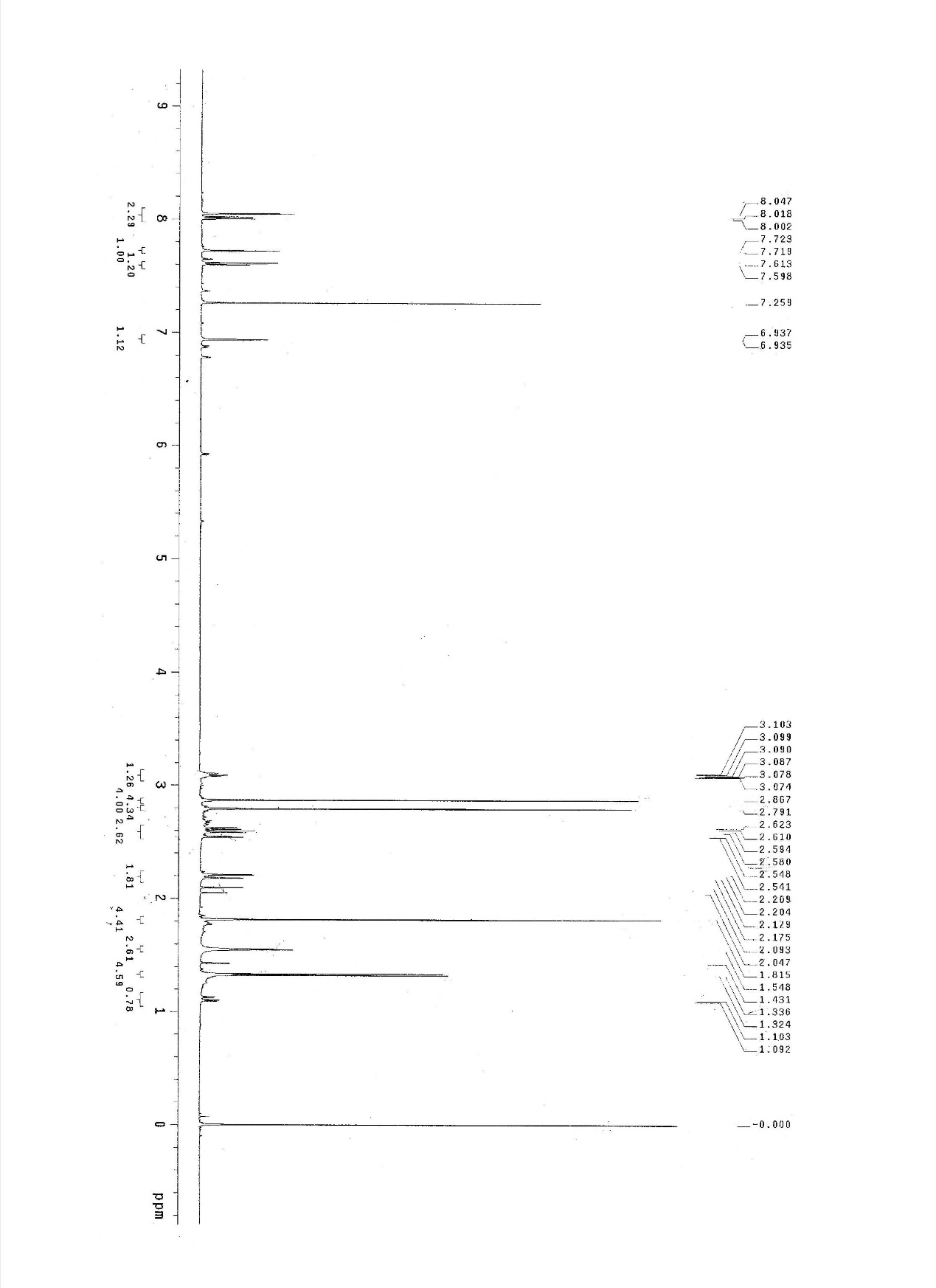


**Figure S2.** ^13^C NMR and DEPT spectra of caesalpinbondin A (**1**) in CDCl_3_


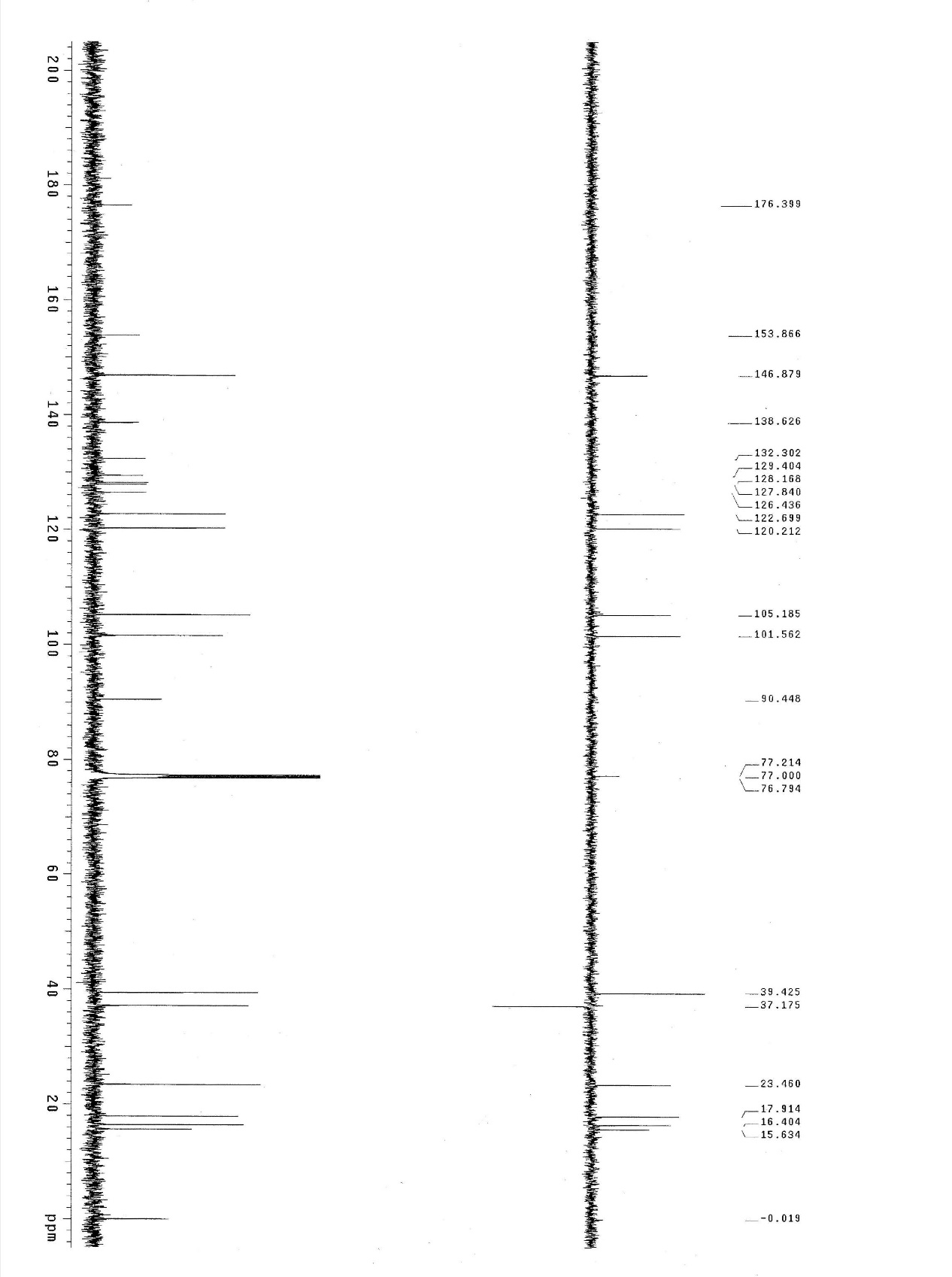


**Figure S3.** HSQC spectrum of caesalpinbondin A (**1**) in CDCl_3_


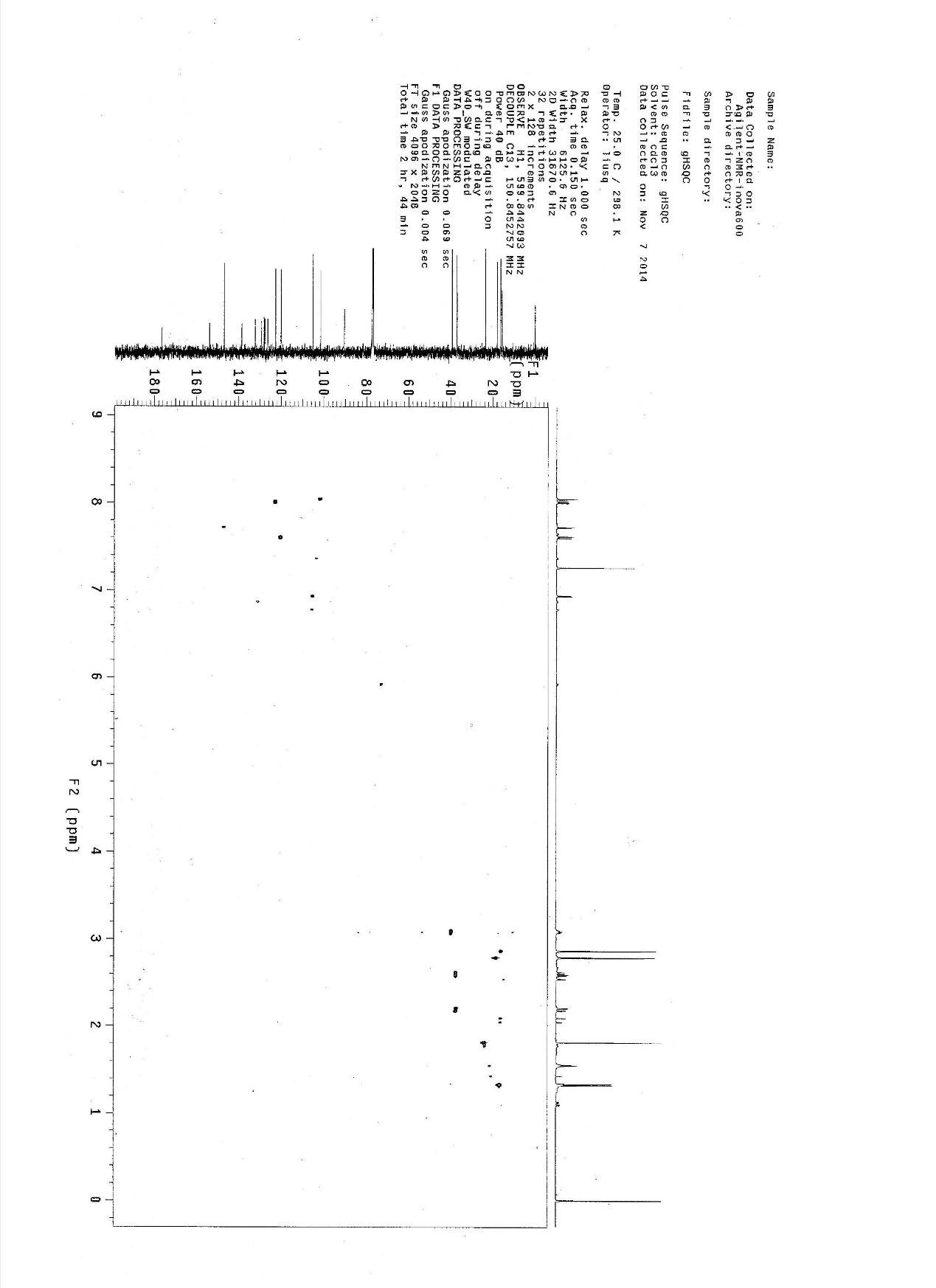


**Figure S4.** HMBC spectrum of caesalpinbondin A (**1**) in CDCl_3_


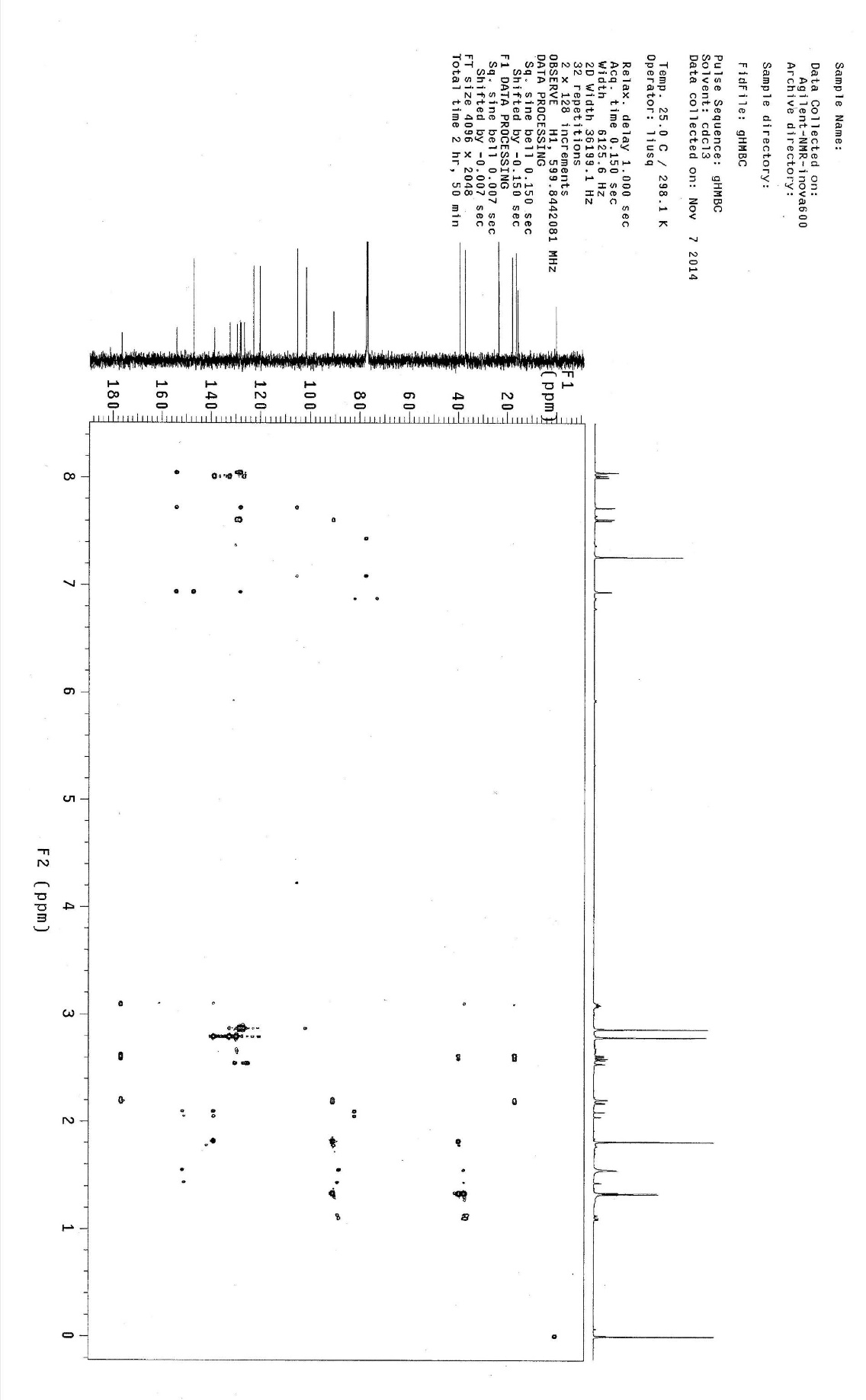


**Figure S5.** ^1^H-^1^H COSY spectrum of caesalpinbondin A (**1**) in CDCl_3_


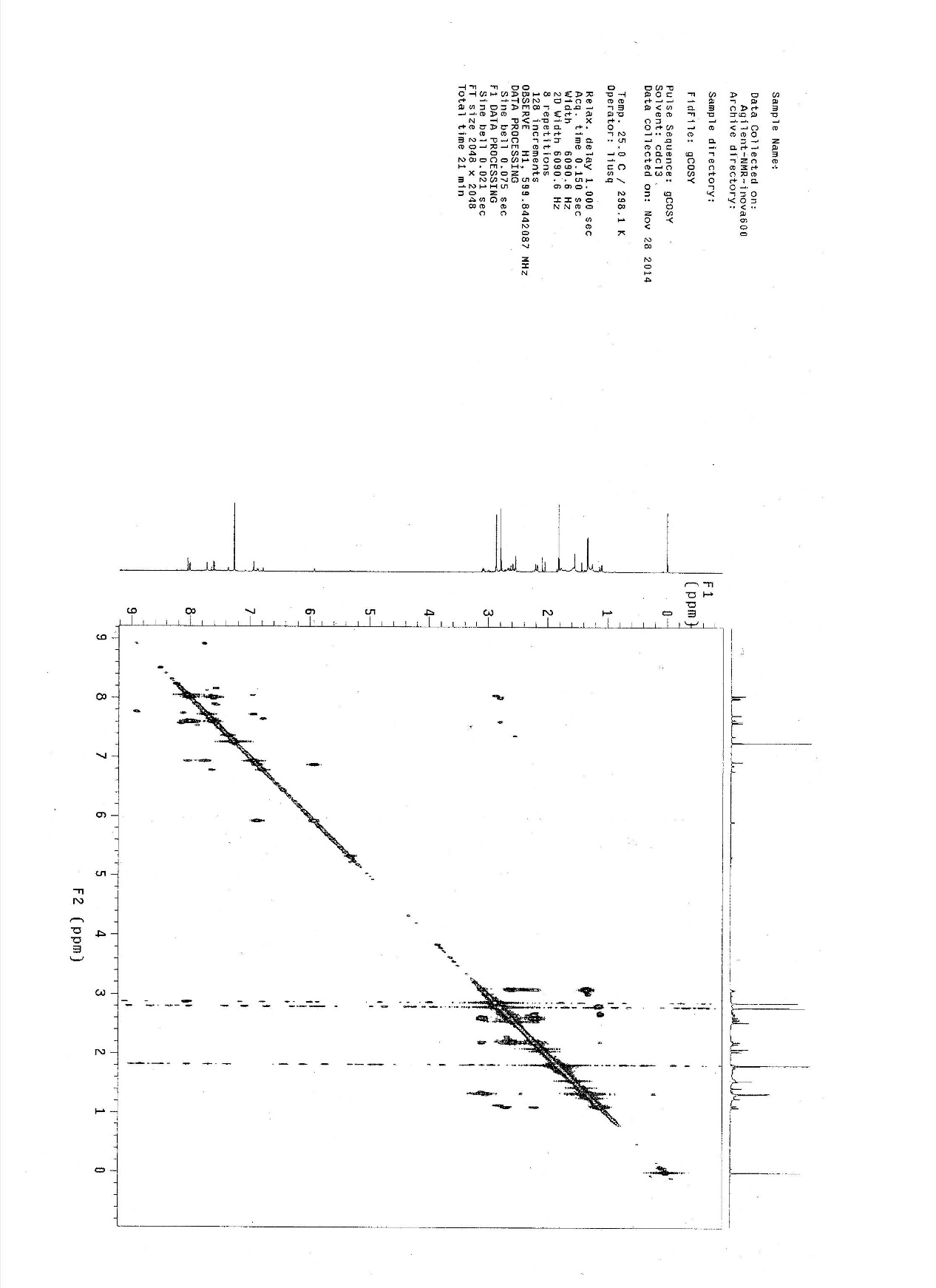


**Figure S6.** NOESY spectrum of caesalpinbondin A (**1**) in CDCl_3_


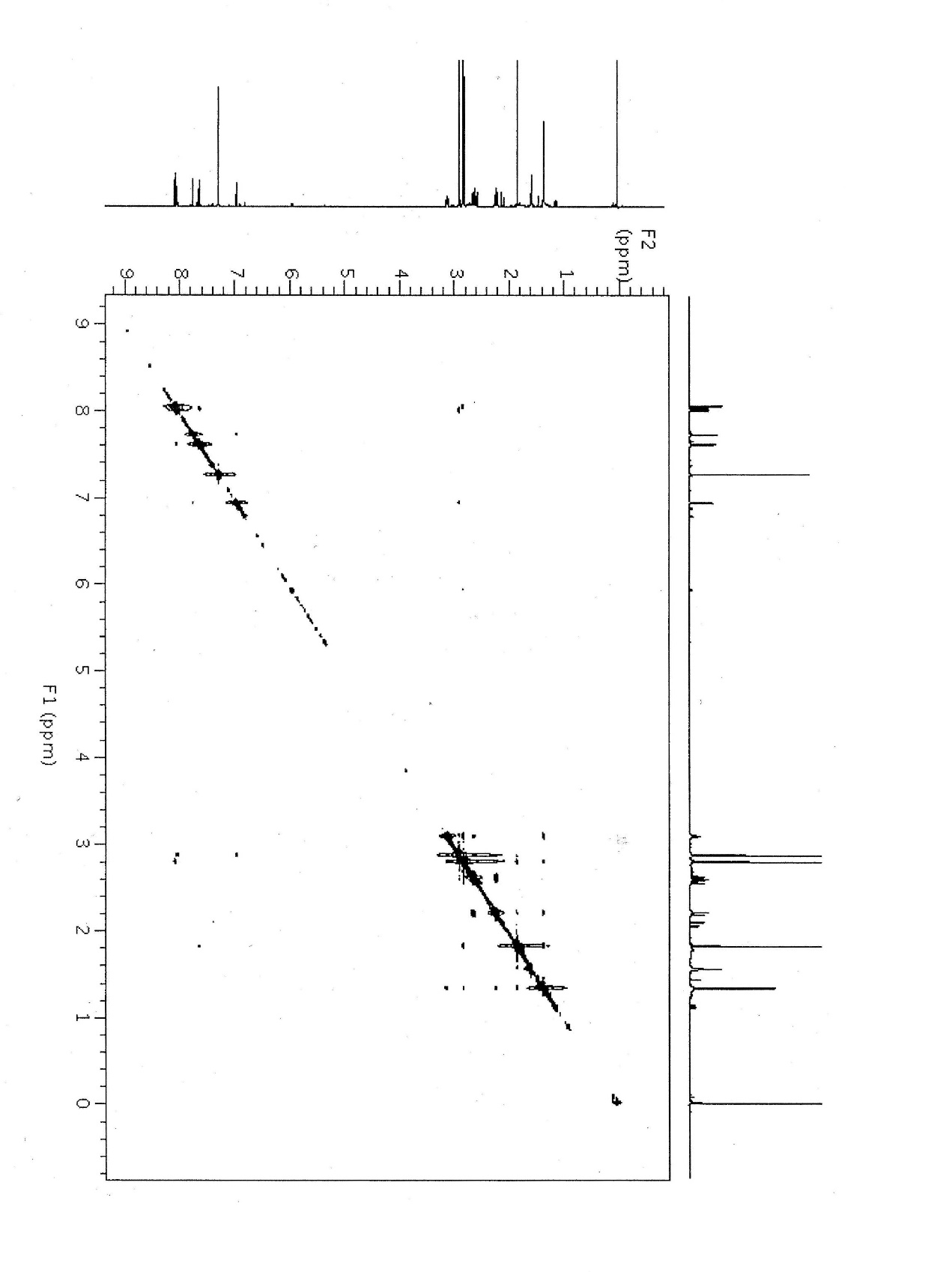


**Figure S7.** HRESIMS spectrum of caesalpinbondin A


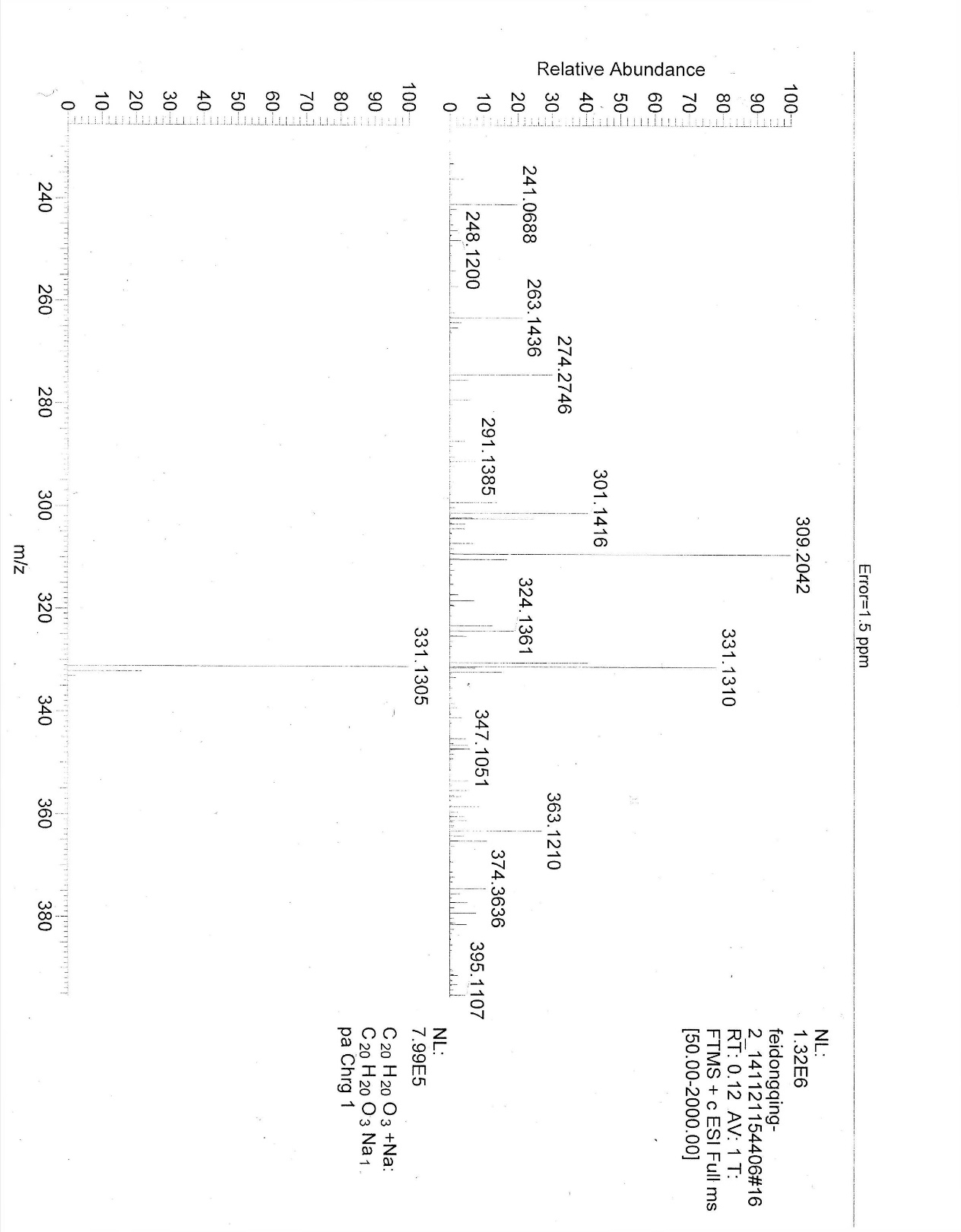


**Figure S8.** IR spectrum of caesalpinbondin A


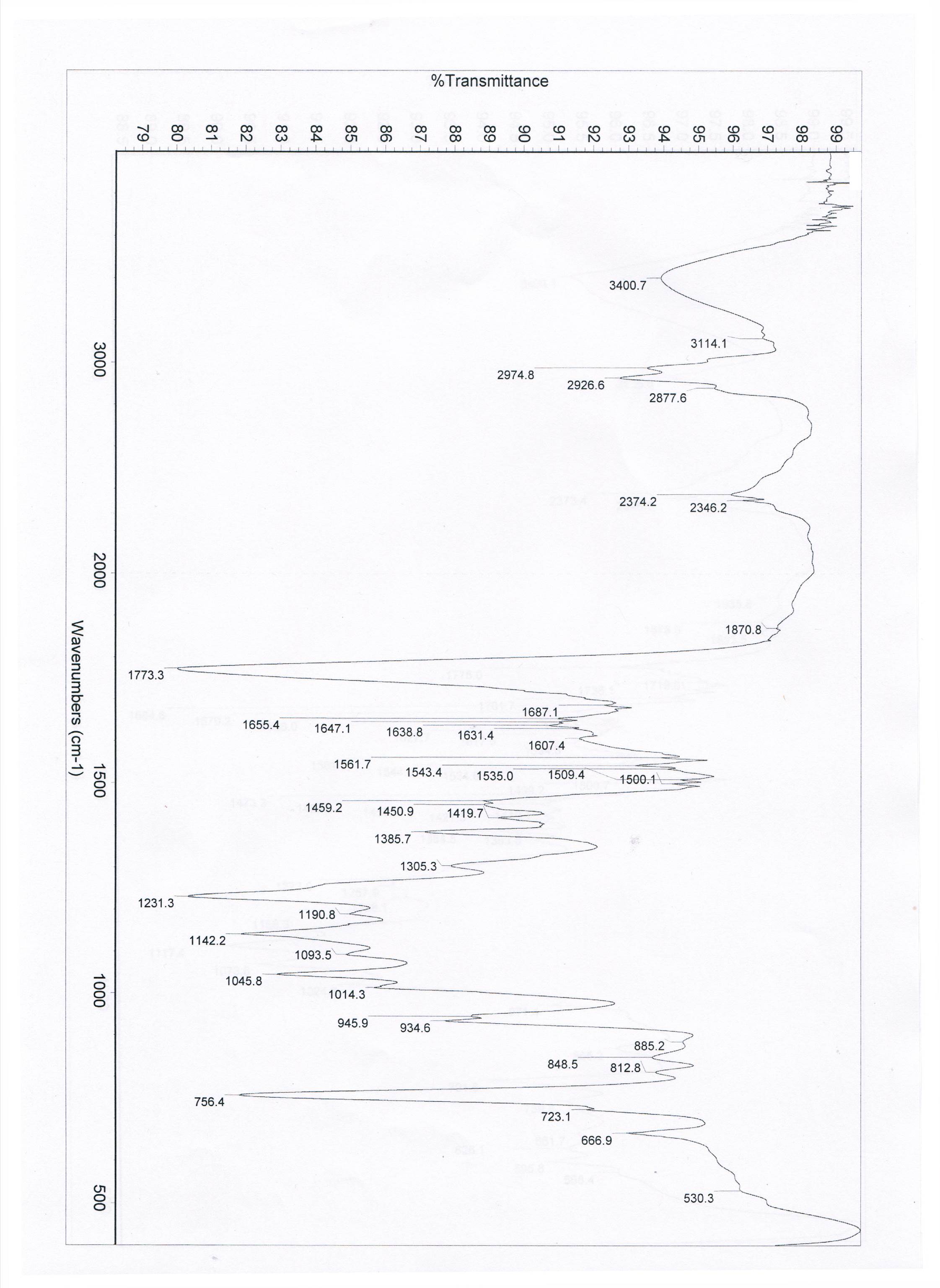


**Figure S9.** UV spectrum of caesalpinbondin A (MeOH)

**Figure S10.** Experimental ECD spectrum of caesalpinbondin A (MeOH)

**Figure S11.** Energy lowest conformers and populations of (3*R*,4*R*)-**1** and optimized coordinates (method: B3LYP/6-31G)


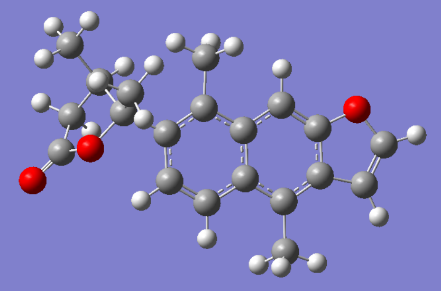

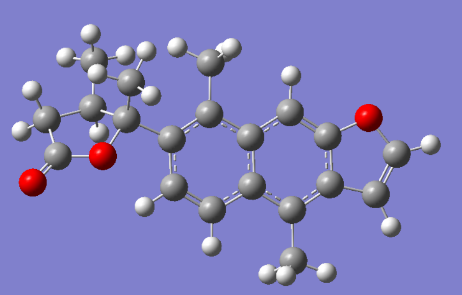

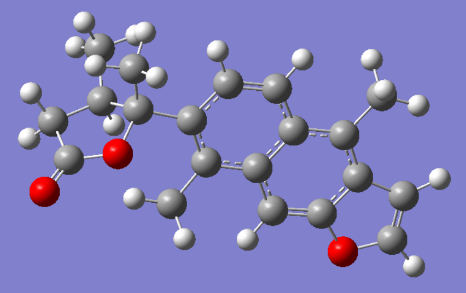


(3*R*,4*R*)-**1_con1**: 86.42 % (3*R*,4*R*)-**1_con2**: 0.58 % (3*R*,4*R*)-**1_con3**: 13.00 %

Standard orientation of (3*R*,4*R*)-**1_con1**:

---------------------------------------------------------------------

Center Atomic Atomic Coordinates (Angstroms)

Number Number Type X Y Z

---------------------------------------------------------------------

1 6 0 1.181548 0.040872 0.426171

2 6 0 0.821864 1.415706 0.518477

3 6 0 -0.478363 1.835825 0.411576

4 6 0 -1.539275 0.913459 0.205484

5 6 0 -1.196081 -0.488520 0.130640

6 6 0 0.179021 -0.911496 0.251609

7 6 0 -2.891066 1.364599 0.083967

8 6 0 -3.875406 0.403031 -0.107565

9 6 0 -3.514480 -0.969604 -0.176531

10 6 0 -2.233494 -1.441737 -0.066180

11 6 0 -5.315862 0.422472 -0.273802

12 6 0 -5.696553 -0.866385 -0.422646

13 8 0 -4.634146 -1.743644 -0.370034

14 6 0 0.438121 -2.405268 0.189785

15 6 0 -3.241224 2.831043 0.159514

16 6 0 2.676299 -0.326407 0.565162

17 6 0 3.244827 -1.021964 -0.726142

18 6 0 3.628239 0.191889 -1.587731

19 6 0 3.990768 1.253425 -0.573320

20 8 0 3.463863 0.917386 0.630483

21 8 0 4.634560 2.267822 -0.721842

22 6 0 4.448278 -1.940900 -0.480449

23 6 0 2.960059 -1.003663 1.911109

24 1 0 1.599031 2.150552 0.685927

25 1 0 -0.697491 2.895403 0.488194

26 1 0 -2.037033 -2.503606 -0.131851

27 1 0 -5.972767 1.280498 -0.280163

28 1 0 -6.661156 -1.328843 -0.572312

29 1 0 0.117491 -2.824845 -0.771851

30 1 0 1.483011 -2.673884 0.322905

31 1 0 -0.127171 -2.929218 0.969941

32 1 0 -2.730214 3.410230 -0.619313

33 1 0 -4.315531 2.985966 0.034378

34 1 0 -2.953515 3.267465 1.123993

35 1 0 2.458446 -1.594845 -1.217496

36 1 0 4.456376 0.012596 -2.277520

37 1 0 2.778701 0.561653 -2.175332

38 1 0 4.808557 -2.334353 -1.437045

39 1 0 5.280666 -1.408426 -0.005483

40 1 0 4.187822 -2.794407 0.153582

41 1 0 2.450444 -1.964280 2.002673

42 1 0 4.033158 -1.162379 2.045246

43 1 0 2.603633 -0.356055 2.717902

---------------------------------------------------------------------

Standard orientation of (3*R*,4*R*)-**1_con2**:

---------------------------------------------------------------------

Center Atomic Atomic Coordinates (Angstroms)

Number Number Type X Y Z

---------------------------------------------------------------------

1 6 0 -1.134546 0.026271 -0.199858

2 6 0 -0.809282 1.403753 -0.011834

3 6 0 0.479784 1.843073 0.125568

4 6 0 1.577168 0.942785 0.067662

5 6 0 1.272225 -0.458921 -0.100463

6 6 0 -0.100065 -0.908958 -0.199266

7 6 0 2.921934 1.416689 0.171867

8 6 0 3.942842 0.477380 0.094131

9 6 0 3.621450 -0.894603 -0.082344

10 6 0 2.347072 -1.388092 -0.177577

11 6 0 5.391265 0.522292 0.142883

12 6 0 5.813780 -0.754053 -0.002152

13 8 0 4.771793 -1.645751 -0.142516

14 6 0 -0.301727 -2.409832 -0.275667

15 6 0 3.228503 2.883546 0.354560

16 6 0 -2.636052 -0.302237 -0.361560

17 6 0 -3.352446 -0.644405 0.995006

18 6 0 -4.747670 -0.037033 0.800603

19 6 0 -4.505792 1.117611 -0.144958

20 8 0 -3.300007 0.966986 -0.742902

21 8 0 -5.219491 2.064464 -0.392060

22 6 0 -3.379560 -2.102866 1.452339

23 6 0 -2.975472 -1.230476 -1.539387

24 1 0 -1.611352 2.129051 0.013225

25 1 0 0.662181 2.902812 0.268257

26 1 0 2.187373 -2.448147 -0.321247

27 1 0 6.025508 1.388329 0.267336

28 1 0 6.798445 -1.197341 -0.027949

29 1 0 0.294164 -2.913752 0.492403

30 1 0 -1.329962 -2.712222 -0.111574

31 1 0 0.014402 -2.815935 -1.245388

32 1 0 2.846595 3.484991 -0.479549

33 1 0 2.774874 3.281779 1.270491

34 1 0 4.305778 3.054733 0.418958

35 1 0 -2.838730 -0.051621 1.761838

36 1 0 -5.442551 -0.741759 0.324721

37 1 0 -5.216133 0.310095 1.724868

38 1 0 -4.003826 -2.188251 2.348609

39 1 0 -3.806957 -2.763303 0.689554

40 1 0 -2.384143 -2.470021 1.715536

41 1 0 -4.061295 -1.286452 -1.667281

42 1 0 -2.548889 -0.813827 -2.456590

43 1 0 -2.604314 -2.246302 -1.423713

---------------------------------------------------------------------

Standard orientation of (3*R*,4*R*)-**1_con3**:

---------------------------------------------------------------------

Center Atomic Atomic Coordinates (Angstroms)

Number Number Type X Y Z

---------------------------------------------------------------------

1 6 0 1.132157 0.596449 -0.221466

2 6 0 0.539380 1.884632 -0.373792

3 6 0 -0.816488 2.080755 -0.357046

4 6 0 -1.716192 0.995515 -0.190655

5 6 0 -1.142130 -0.321210 -0.035713

6 6 0 0.290985 -0.506020 -0.049242

7 6 0 -3.130211 1.209850 -0.177031

8 6 0 -3.946626 0.099291 -0.004957

9 6 0 -3.361088 -1.186450 0.147142

10 6 0 -2.014192 -1.432794 0.136870

11 6 0 -5.376828 -0.126540 0.070288

12 6 0 -5.540035 -1.456469 0.252402

13 8 0 -4.342300 -2.136804 0.304655

14 6 0 0.778273 -1.931047 0.130029

15 6 0 -3.719083 2.589772 -0.343426

16 6 0 2.668883 0.523831 -0.208817

17 6 0 3.263742 0.099449 1.177068

18 6 0 4.526476 -0.672998 0.770795

19 6 0 4.204245 -1.199424 -0.616441

20 8 0 3.112079 -0.563218 -1.111216

21 8 0 4.776283 -2.058316 -1.250063

22 6 0 3.481944 1.231989 2.180185

23 6 0 3.355020 1.778610 -0.769257

24 1 0 1.170422 2.752974 -0.508426

25 1 0 -1.204058 3.086725 -0.475712

26 1 0 -1.646868 -2.442609 0.259644

27 1 0 -6.169762 0.604078 -0.001420

28 1 0 -6.419138 -2.074325 0.362708

29 1 0 0.362356 -2.583203 -0.647441

30 1 0 1.855470 -2.030458 0.075978

31 1 0 0.451240 -2.336926 1.095127

32 1 0 -3.432116 3.039460 -1.301895

33 1 0 -4.810689 2.558452 -0.307291

34 1 0 -3.381082 3.273225 0.445229

35 1 0 2.561528 -0.610466 1.624757

36 1 0 5.408651 -0.023354 0.702353

37 1 0 4.783605 -1.499115 1.438636

38 1 0 3.808057 0.820254 3.141203

39 1 0 4.248792 1.937601 1.844337

40 1 0 2.554186 1.788382 2.354748

41 1 0 4.435339 1.614175 -0.821392

42 1 0 2.997898 1.987966 -1.781421

43 1 0 3.188281 2.660718 -0.148355

---------------------------------------------------------------------
